# Aortic Valve Calcium in Relation to Subclinical Cardiac Dysfunction and Risk of Heart Failure

**DOI:** 10.1161/CIRCIMAGING.122.014323

**Published:** 2023-03-07

**Authors:** Fang Zhu, Yannick Kaiser, Eric Boersma, Daniel Bos, Maryam Kavousi

**Affiliations:** 1 Department of Epidemiology, Erasmus MC, University Medical Center Rotterdam, The Netherlands (F.Z., Y.K., D.B., M.K.).; 2 Department of Vascular Medicine, Amsterdam Cardiovascular Sciences, Amsterdam UMC, University of Amsterdam, The Netherlands (Y.K.).; 3 Department of Cardiology, Erasmus MC, University Medical Center Rotterdam, The Netherlands (E.B.).; 4 Department of Radiology and Nuclear Medicine, Erasmus MC, University Medical Center Rotterdam, The Netherlands (D.B.).

**Keywords:** aortic valve calcium, cardiac computed tomography, cardiac dysfunction, heart failure, vascular calcification

## Abstract

The link between (mild) aortic valve calcium (AVC) with subclinical cardiac dysfunction and with risk of heart failure (HF) remains unclear. This research aims to determine the association of computed tomography-assessed AVC with echocardiographic measurements of cardiac dysfunction, and with HF in the general population.

**Methods:**

We included 2348 participants of the Rotterdam Study cohort (mean age 68.5 years, 52% women), who had AVC measurement between 2003 and 2006, and without history of HF at baseline. Linear regression models were used to explore relationship between AVC and echocardiographic measures at baseline. Participants were followed until December 2016. Fine and Gray subdistribution hazard models were used to assess the association of AVC with incident HF, accounting for death as a competing risk.

**Results:**

The presence of AVC or greater AVC were associated with larger mean left ventricular mass and larger mean left atrial size. In particular, AVC ≥800 showed a strong association (body surface area indexed left ventricular mass, β coefficient: 22.01; left atrium diameter, β coefficient: 0.17). During a median of 9.8 years follow-up, 182 incident HF cases were identified. After accounting for death events and adjusting for cardiovascular risk factors, one-unit larger log (AVC+1) was associated with a 10% increase in the subdistribution hazard of HF (subdistribution hazard ratio, 1.10 [95% CI, 1.03–1.18]), but the presence of AVC was not significantly associated with HF risk in fully adjusted models. Compared with the AVC=0, AVC between 300 and 799 (subdistribution hazard ratio, 2.36 [95% CI, 1.32–4.19]) and AVC ≥800 (subdistribution hazard ratio, 2.54 [95% CI, 1.31–4.90]) were associated with a high risk of HF.

**Conclusions:**

Presence and high levels of AVC were associated with markers of left ventricular structure, independent of traditional cardiovascular risk factors. Larger computed tomography-assessed AVC is an indicative of increased risk for the development of HF.

**Clinical Perspective**

Aortic valve calcium (AVC) is common among older adults. However, the link between (mild) AVC with subclinical cardiac dysfunction and with risk of heart failure (HF) remains unclear. In this study consisting 2348 participants from the Rotterdam Study and with a 9.8 years follow-up, we found that the presence of AVC and higher AVC were associated with larger left ventricular mass and left atrium size. AVC >300 AU was associated with higher HF risk and AVC ≥800 AU was significantly related with left ventricular structure remodeling. These findings suggest that AVC load could be related with subclinical changes in left ventricular structure and risk of HF, which may carry the premise for understanding the pathogenesis of AVC in cardiac dysfunction and HF.

**See Editorial by Sharma and Morrison**

Heart failure (HF) is a complex clinical syndrome induced by structural or functional cardiac abnormalities, resulting in elevated intracardiac pressures and inadequate cardiac output.^1^ Currently, the incidence of HF in Europe is about 5/1000 person-years in adults, and the overall incidence is increasing due to aging.^1^ Valvular heart disease has been described as the next cardiac epidemic,^2^ which is considered a potential contributor of HF.^3,4^

Aortic valve calcium (AVC) manifests as ectopic calcium and phosphate deposits on the aortic valve. AVC leads to valve stiffness, reduced leaflet deviation, and progressive valve narrowing.^5,6^ Although it was previously considered a harmless degenerative condition as a natural consequence of aging, emerging evidence suggests that AVC is associated with adverse cardiovascular outcomes and all-cause mortality.^7,8^ The aortic valve maintains optimal coronary perfusion and controls the laminar flow of blood into the vascular system.^9^ Disturbed flow from calcified valves exerts downstream effects on the vascular system such as vascular calcification^10,11^ and potential left ventricular dysfunction in response to increased afterload chronically.^12^ Consequently, a link between (mild) AVC with subclinical cardiac dysfunction and with new-onset HF seems plausible.

Identification of the underlying risk factors of subclinical cardiac dysfunction creates opportunities to ensure early prevention or optimal subsequent treatment. However, comprehensive assessment of the impact of AVC on cardiac function in general population is lacking. Using data from the prospective population-based Rotterdam Study, we investigated the associations of AVC, detected on computed tomography (CT) scans, with echocardiographic parameters of cardiac structure and function and with new-onset HF in the general population.

## Methods

The authors declare that all supporting data are available from the data manager of the Rotterdam Study, Frank J.A. van Rooij (f.vanrooij@erasmusmc.nl) upon reasonable request.

### Study Population

This study was embedded within the Rotterdam Study, an ongoing prospective population-based cohort in adults aged ≥45 years.^13^ The participants underwent extensively examined at baseline and subsequent follow-up examinations taking place every 3 to 4 years.

**Figure 1.**
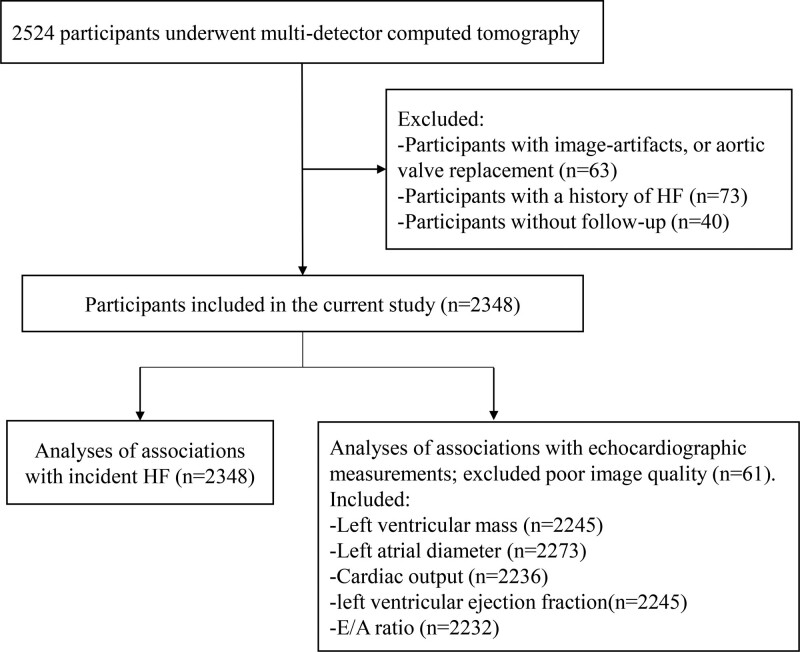
**Flow chart of the study population.** HF indicates heart failure.

The current study is focused on a random sample of 3229 participants whom between 2003 and 2006, during their visit to the research center, were invited to undergo multidetector CT for the visualization of arterial calcification of which AVC was part as well. In total, 2524 participants were scanned (response rate, 78%). Due to image-artifacts, or aortic valve replacement, 2461 participants were left with a usable AVC measurement. We further excluded participants with a history of HF (n=73) or those without follow-up (n=40). As a result, 2348 individuals were included. Regarding echocardiographic assessments, we excluded 61 participants due to poor image quality. Finally, information was available on left ventricular (LV) mass for 2245 participants, on left atrium diameter (LAD) for 2273 participants, on cardiac output for 2236 participants, left ventricular ejection fraction (LVEF) for 2245 participants, and on E/A ratio for 2232 participants. Figure 1 displays the selection of the study population.

The Rotterdam Study has been approved by the Medical Ethics Committee of the Erasmus MC (registration number MEC 02.1015) and by the Dutch Ministry of Health, Welfare, and Sport (Population Screening Act WBO, license number 1071272-159521-PG). All participants provided written informed consent to participate in the study and to have their information obtained information from their treating physicians.

### Assessment of AVC and Coronary Artery Calcium

Noncontrast, ECG-gated, cardiac CT images were obtained using 16-slice or 64-slice multidetector CT scanners (Somatom Sensation 16 or 64; Siemens, Forchheim, Germany), ranging from the apex of the heart to the tracheal bifurcation. Details on the scan parameters were previously given.^14^ All calcified lesions (Hounsfield Units >130) located on the aortic valve cusps including the base of the cusps and the aortic valve annulus were quantified. AVC and coronary artery calcium scores were quantified using Agatston methodology by experienced readers who were blinded to the clinical data.

### Assessment of Echocardiographic Parameters

At baseline, participants underwent both AVC measurement and echocardiographic measurements. The average time interval between these 2 measures was 3.8 months. Echocardiographic parameters were measured without knowledge on participants’ AVC status from the CT scan. The echocardiogram for each participant was obtained by certified, experienced echocardiographers according to a standardized protocol.^15^ Echocardiograms were performed with the ultrasonography system (AU3 Partner, Esaote Biomedica, with a 3.5/2.5 MHz transducer) and Acuson Cypress (with a 3V2c transducer).^15^ The interreader and intrareader agreements were good.^16^ Structural parameters were measured in the parasternal long-axis view with the use of M-mode with 2-dimensional guidance, including LAD, left ventricular end-diastolic and end-systolic diameter, end-diastolic interventricular septum thickness, and end-diastolic left ventricular posterior wall thickness. LV mass in grams was calculated as 0.8×(1.04×[left ventricular end-diastolic diameter+interventricular septum thickness+left ventricular posterior wall thickness]^3^−left ventricular end-diastolic diameter^3^)+0.6. End-diastolic volume and end-systolic volume were computed by summation-of-disks method to derive LVEF ([end-diastolic volume−end-systolic volume]/end-diastolic volume). Cardiac output (L/min) was calculated as (end-diastolic volume−end-systolic volume)×heart rate. Pulsed Doppler recordings of the transmittal filling velocity were performed in the apical 4-chamber view, with the sample volume placed in the mitral valve orifice near the tips of the leaflets. Doppler peak E and peak A velocities were averaged over 3 cycles. E/A ratio was computed by dividing the Doppler peak E velocity by the Doppler peak A velocity. In addition, the aortic valve stenosis (AVS) was defined as the presence of AVC on echocardiography and aortic flow velocity >2.5 m/sec (under 60 years) or >3 m/sec (>60 years). Information regarding the presence of AVS was recorded in the latter 2 subsequent follow-up visits (2009–2012 and 2014–2016).

### Incident HF

The Rotterdam Study participants are monitored continuously for the incident cardiovascular events through automated linkage with data from their general practitioner.^17^ Incident HF was defined as a combination of the presence of typical symptoms or signs of HF based on the European Society of Cardiology guideline and confirmed by the medical specialists.^17^ Subjects were followed until the occurrence of HF, death, or end of follow-up time (December 2016), whichever came first.

### Assessment of Mortality

Information on vital status of participants of the Rotterdam Study is obtained from municipal health authorities in Rotterdam and updated monthly for all-cause mortality.

### Assessment of Cardiovascular Risk Factors

History of coronary heart disease (CHD) was verified from the medical records of the general practitioner, and was defined as myocardial infarction and revascularization. Atrial fibrillation was defined in accordance with the European Society of Cardiology guideline.^18^ Blood pressure was measured using a random-zero sphygmomanometer on the right arm twice and the average of the 2 measurements was used. Diabetes was defined as a fasting glucose level ≥7 mmol/L or nonfasting glucose level ≥11.1 mmol/L, or the use of glucose-lowering medication. Serum total cholesterol and high-density lipoprotein cholesterol were assessed using comparable enzymatic procedures. Trained nonmedical interviewers administered a standardized questionnaire to obtain information on medical history at the baseline, smoking status, and pharmacy prescription records. Smoking status was defined as current versus noncurrent smoking. Height and weight were measured and body mass index was calculated as weight (kg)/(height [m])^2^. Body surface area (BSA) was calculated by using the Mosteller formula: BSA=0.016667×weight (kg)^0.5^×height (cm)^0.5^.

### Statistical Analysis

Baseline characteristics of the study population were presented as mean (SD) for normally distributed variables, median (interquartile range) for skewed variables, and number (percentage) for categorical variables. We used Student *t* test for ANOVA for continuous variables with normal distribution, Mann-Whitney *U* test for continuous variables with skewed distribution, and Pearson χ^2^ test for categorical variables to compare baseline characteristics between participants with and without the presence of AVC.

The levels of AVC were investigated on 3 scales, including the naturally log-transformed continuous AVC [log AVC+1], presence of AVC (AVC=0 versus AVC >0), and categorical AVC levels (=0, 1–99, 100–299, 300–799, ≥800; Agatston units [AU]). We used multivariable linear regression to determine the cross-sectional associations between AVC and echocardiographic parameters. AVC was set as an exposure variable, and echocardiographic measures were set as outcomes. Next, we constructed crude (unadjusted) cumulative incidence plots to show the incidence of HF among categorical AVC groups, and gray’s test was used to test the difference of cumulative incidence curves between the groups.^19^ Fine and Gray subdistribution hazard models accounting for the competing risk of death were used to assess the association of AVC with HF. In addition, results of Kaplan-Meier curves and Cox proportional hazard models, which did not prespecify any adjustment for the competing risk of death, are presented in the Supplemental Material (Figure S1 and Table S1). All analyses were adjusted for potential confounding by age and sex (model 1), and additional body mass index, systolic blood pressure, diabetes, current smoking, total and high-density lipoprotein cholesterol, statins therapy, blood pressure-lowering medication, history of CHD, history of atrial fibrillation, and categorical coronary artery calcium (0, 1–100, 101–300, and >300; model 2). To determine whether associations would be affected by sex, interaction effects between sex and AVC were tested in all models.

In sensitivity analyses, we repeated the analyses regarding AVC and incident HF by (1) excluding those with CHD or atrial fibrillation at baseline, (2) excluding those with presence of AVS, and (3) excluding those who developed CHD during follow up and prior to HF onset. The maximum missing of covariates was up to 2.6% of the participants. Missing values in covariates were imputed using a multiple imputation method. Five imputed datasets were generated and summarized estimates were calculated using the MICE package in R. Two-sided *P* value was considered significant at *P*<0.05. Statistical analyses were performed with the use of R version 4.0.3 (https://www.r-project.org).

## Results

### Baseline Characteristics

The study included 2348 participants with a mean (±SD) age of 68.5±6.6 years, 52% of whom were women (Table 1). The prevalence of AVC was 31.8% (n=747), 474 participants had AVC score between 1 and 99, 165 participants had AVC between 100 and 299, 56 participants had AVC between 300 and 799, and 52 participants had AVC ≥800. Participants with AVC >0 had higher LV mass, LAD, LVEF, and E/A ratio.

**Table 1.**
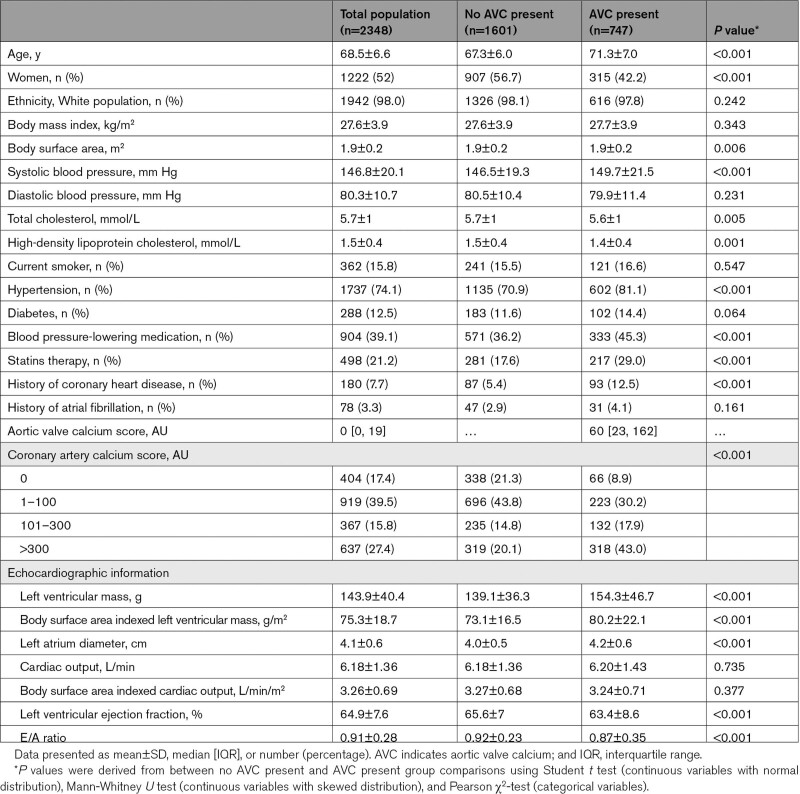
Baseline Characteristics of the Study Population, Stratified Based on the Presence of Aortic Valve Calcium

### AVC and Echocardiographic Parameters of Cardiac Structure and Function

The presence of AVC was significantly associated with larger BSA-indexed LV mass, larger LAD, and worse LVEF in both age- and sex-adjusted model and fully adjusted models (Table 2). Among AVC categories, AVC ≥800 was largely associated with BSA-indexed LV mass (β, 22.01 [95% CI, 17.03–26.99]; model 2) and LAD (β, 0.17 [95% CI, 0.03–0.31]; model 2), compared with AVC=0 (Table S2). One-unit higher log (AVC+1) was associated with 1.13 g/m^2^ (β, 1.13 [95% CI, 0.77–1.50]; model 2) higher mean BSA-indexed LV mass, and 0.01 mm (β, 0.01 [95% CI, 0.01–0.02]; model 2) larger LAD. For LVEF, AVC presence was associated with worse LVEF (β, −0.68 [95% CI, −1.36 to −0.01]; model 2) compared with AVC=0, while higher AVC did not show significant association with LVEF. Besides, there were no significant associations between the presence of AVC or higher AVC and cardiac output and E/A ratio.

**Table 2.**
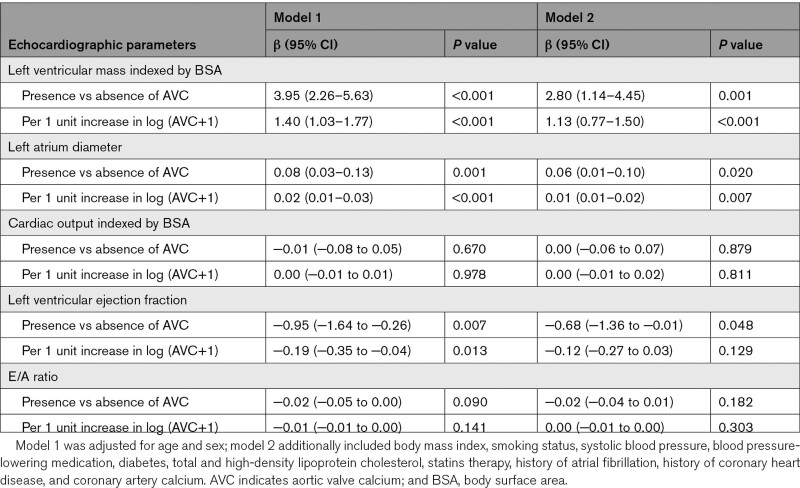
Multivariable Linear Regression Analyses of the Association Between Aortic Valve Calcium and Echocardiographic Parameters of Cardiac Structure and Function

### AVC, AVS, and Incident HF

During a median follow-up of 9.8 years, a total of 182 participants were diagnosed with HF. The HF incident rate in AVC present group and non-AVC present group were 12.6% (94 incident cases) and 5.5% (88 incident cases), respectively. At baseline, 14 participants were identified as having AVS, and 23 participants subsequently had AVS during follow-up visits. HF occurred in 11 of total of 37 patients with AVS (29.7%), compared with 7.4% (171/2311) cumulative incident among participants with no AVS.

**Figure 2.**
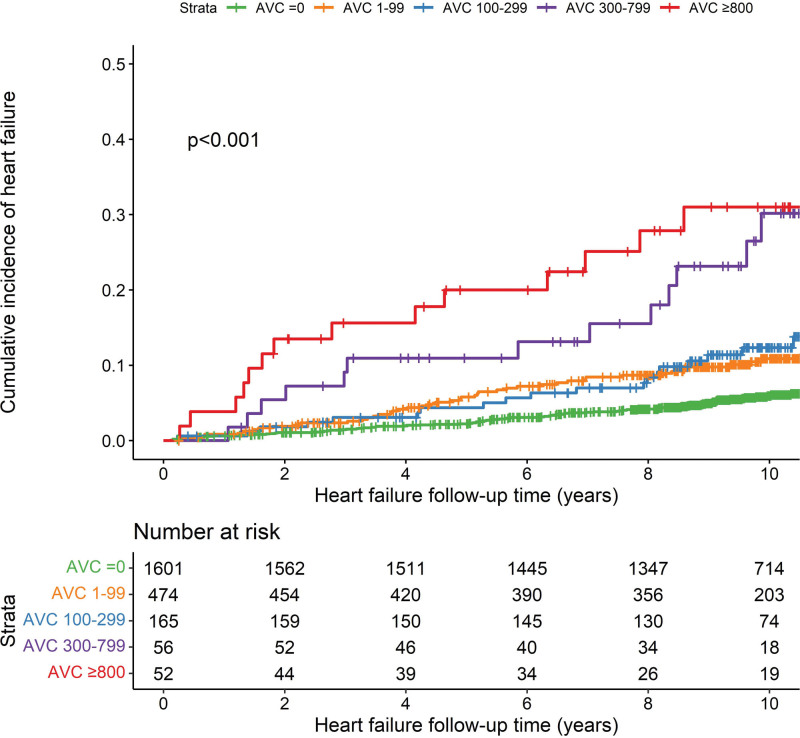
**Cumulative incidence curves for incident heart failure according to the aortic valve calcium categories.** AVC indicates aortic valve calcium.

Cumulative incidence curves showed that incident HF significantly differed among participants with different AVC levels (*P*<0.001; Figure 2). Table 3 displays adjusted subdistribution hazard ratios (sHRs) for the association of AVC with incident HF. After accounting for death events and adjusting for potential confounding, one-unit larger log (AVC+1) was associated with a 10% increase in the subdistribution hazard of HF (sHR, 1.10 [95% CI, 1.03–1.18]), but the presence of AVC was not significantly associated with HF risk in fully adjusted models (sHR, 1.34 [95% CI, 0.97–1.86]). For multicategorical AVC levels, compared with the reference category (AVC=0), AVC between 300 and 799 (sHR, 2.36 [95% CI, 1.32–4.19]) and AVC ≥800 HF (sHR, 2.54 [95% CI, 1.31–4.90]) were significantly associated with increased HF risk. No significant sex interaction with AVC was detected.

**Table 3.**
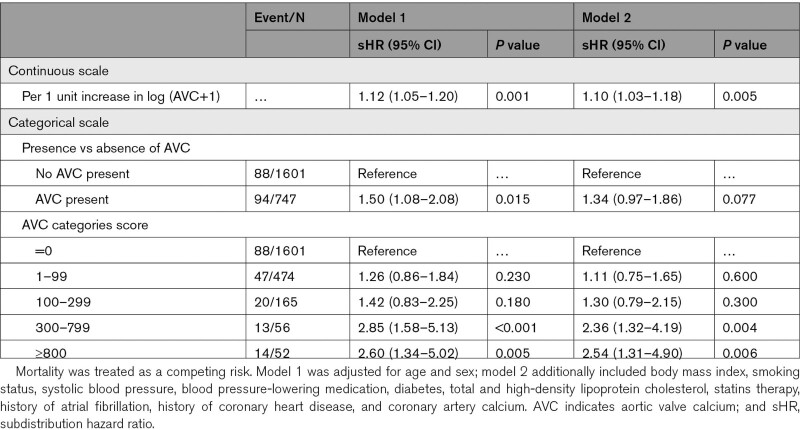
Multivariable Competing Risk Regression Analyses of the Association Between Aortic Valve Calcium and Incident Heart Failure

### Competing Risk of Mortality

A total of 556 (23.7%) participants died during follow-up. AVC did not show any significant associations with risk of mortality after adjusting for traditional cardiovascular risk factors (Table S3).

### Sensitivity Analysis

The presence of AVC and higher AVC scores were independently associated with HF risk among participants free of CHD or atrial fibrillation at baseline (Table S4). After excluding patients with AVS, the association between AVC and incident HF attenuated, while AVC between 300 and 799 was significantly associated with higher HF risk (sHR, 2.25 [95% CI, 1.19–4.22]; Table S5). After excluding those who developed CHD during follow-up and prior to HF onset (n=33), the association between AVC and incident HF attenuated, while AVC ≥800 was associated with higher HF risk (sHR, 2.30 [95% CI, 1.19–4.88]; Table S6).

## Discussion

Using data from the prospective population-based Rotterdam Study cohort, we found that the presence of AVC and higher AVC scores were associated with increased LV mass and LAD, independent of traditional cardiovascular risk factors. Moreover, higher AVC scores (ie, >300 AU) was associated with a higher risk of new-onset HF.

Nowadays, AVC is not routinely assessed in clinical practice and the clinical consequences in individuals in the early stages of calcification of aortic valve remain unclear. The advent of automated quantification algorithms, capable of assessing AVC even on noncardiac CT scans, makes the routine imaging assessment AVC more feasible in the foreseeable future. In the present study, AVC was present among nearly one-third of the individuals, which highlights the importance of determining the clinical relevance of (mild) AVC to cardiac dysfunction. The findings of this study suggest that AVC load could be related with changes in left ventricular structure and increased risk of HF, which may carry the premise for understanding the pathogenesis of AVC in cardiac dysfunction and HF.

Our study shows that the presence of AVC and higher AVC scores were significantly associated with larger LV mass and LAD, and AVC ≥800 AU was a strong risk factor. Higher LV mass has been previously reported to be significantly associated with prevalence, severity, and incidence of AVC.^20^ Khurrami et al^21^ recently found that increasing AVC scores was significantly associated with left atrial dilation and LV hypertrophy among men aged 65 to 74 years with an AVC score >300 AU at baseline. Mechanistically, the shared risk factors and possible common pathophysiological mechanisms could partly account for the close relation between AVC formation and LV remodeling. When a normal valve is chronically exposed to biochemical (ie, hypercholesterolemia and hyperglycemia) or mechanical (ie, blood pressure) stress, the valve leaflets progressively thicken and become fibrotic with evidence of inflammation and calcification.^10,22^ Inappropriate vascular calcification also leads to mineralization of the internal elastic lamina and elastic fibers in the arterial wall, thereby increasing stiffness.^23^ Those risk factors (including hypercholesterolemia, hyperglycemia, and hypertension) and changes (including arterial stiffness) mentioned are major determinants of LV hypertrophy and left atrial enlargement as well.^24,25^ Besides, Gomel et al^10^ discussed that in late-stage calcification of aortic valve, the more rigid and thick calcified valves are impeded to fully open under systolic pressure and fully close under diastolic pressure. This reduced motion lowers the ejection fraction of the valve while also increasing retrograde leakage into the ventricle, which also may contribute to the left ventricular dysfunction in response to the disturbed flow. Our study showed a borderline significant association (*P*=0.048) between AVC presence and LVEF, whereas higher AVC scores were no longer associated with LVEF, indicating that the presence of AVC may be related to LV systolic function. Of note, we only assessed association between echocardiographic parameters and AVC among the baseline population without overt HF, while late changes in LV modeling and dysfunction were not then discernible, implying that we may have underestimated the associations. Moreover, we cannot rule out the possibility of residual or unmeasured confounding.

Although currently only the presence of late-stage AVC, such as AVS, has clinical consequences, AVC presence and increasing AVC load were found to be associated with risk of new-onset HF in our study. Considering that ischemic heart disease is the major underlying pathogenic factor of HF incidence,^26^ we included sensitivity analyses to consider the impact of incident CHD. After excluding those who developed CHD during follow-up and prior to HF onset, the association between AVC and HF attenuated. This suggests that the pathophysiology from AVC to HF development may be partly through the ischemia pathway. Indeed, previous research reported AVC was associated with CHD and other cardiovascular outcomes. Owens et al^7^ investigated 6685 participants free of CVD and demonstrated that subjects with AVC had higher risks of cardiovascular and coronary events compared with those without AVC. Christensen et al^8^ found similar significant independent associations of AVC score with myocardial infarction and cerebrovascular events. This, in combination with our results, underlines the importance of AVC as a marker of subclinical atherosclerosis, as well as structural heart disease.

Aging plays an essential role in AVC formation, LV remodeling, and incident HF, so aging-related changes may promote AVC progression and cardiac remodeling simultaneously, causing potential confounding. However, the associations between AVC presence and severity with echocardiographic parameters and with incident HF were independent of age and other cardiovascular risk factors in our study. We also checked the association of AVC with mortality, and found higher AVC load had limited impact on risk of mortality, suggesting that mortality, a competing risk of HF, did not materially affect the association between AVC and HF.

AVC is the main hallmark of AVS, insights regarding pathophysiological mechanisms that link AVC to cardiac dysfunction can be gained from the link between AVS and LV dysfunction. Chronic LV pressure overload from AVS causes both increases in myocardial muscle and changes in LV geometry as compensatory mechanism to reduce wall stress and maintain cardiac output.^27,28^ When wall stress exceeds the compensating mechanism, LV contractile function declines.^3^ Hence, AVC may affect cardiac function through stenosis of aortic valve. We further did sensitivity analyses by excluding AVS patients, and found that AVC ≥800 lost the significant association with HF, probably due to the exclusion of participants with severe AVC; while AVC load between 300 and 799 was significantly associated with risk of HF, suggesting that AVC burden at mild levels still affect cardiac function.

The strengths of our study include a well-characterized cohort with availability of CT-based AVC quantification and echocardiographic assessments of cardiac structure and function, as well as detailed information on HF status and long follow-up. There are also some limitations. First, the study population comprised the elderly population of white European ancestry. Therefore, our results might not be generalizable to younger populations and other ancestries. Second, we only assessed cross-sectional association between echocardiographic parameters and AVC among the baseline population without overt HF. Longitudinal studies with repeated echocardiographic measurements could provide more information. Third, AVS was only assessed at research center visits and the sequence of AVS and HF occurrences was unclear. However, as the total number of patients with AVS is small, we believe this would not materially affect our findings for AVC. Fourth, we do not have data for a distinction of HF phenotypes to further explore and compare the underlying mechanisms.

## Conclusions

In this prospective study among community-dwelling men and women free of HF, the presence of AVC and high levels of AVC were associated with left ventricular structure and increased risk of new-onset HF. Our results suggest that larger CT-assessed AVC could be an indicative of increased risk for development of HF.

## Article Information

### Acknowledgments

The authors are grateful to the study participants, the staff from the Rotterdam Study, and participating general practitioners and pharmacists.

### Sources of Funding

The Rotterdam Study is supported by Erasmus MC and Erasmus University Rotterdam; the Netherlands Organization for Scientific Research; the Netherlands Organization for Health Research and Development (ZonMw); the Research Institute for Diseases in the Elderly; the Netherlands Genomics Initiative; the Ministry of Education, Culture, and Science; the Ministry of Health, Welfare, and Sports; European Commission; and the Municipality of Rotterdam. F. Zhu is sponsored by Chinese Government Scholarship (202007720001). Dr Kaiser was supported by the Netherlands Heart Foundation CVON 2017-20: generating the best evidence-based pharmaceutical targets for atherosclerosis [GENIUS II]). This project is further supported by the Senior Scientist Grant from the Dutch Heart Foundation (03-004-2021-T050).

### Disclosures

None.

### Supplemental Material

Figure S1

Tables S1–S6

## Supplementary Material
