# Supplementary material for: Aortic Valve Calcium in Relation to Subclinical Cardiac Dysfunction and Risk of Heart Failure
Source: Circ Cardiovasc Imaging. 2023 Mar 7;16(3):e014323. doi: 10.1161/CIRCIMAGING.122.014323 (PMC10026958; doi:10.1161/CIRCIMAGING.122.014323)
Supplement: Supplementary file 1 [file hci-16-e014323-s001.pdf]

SUPPLEMENTAL MATERIAL

Supplemental Figure.

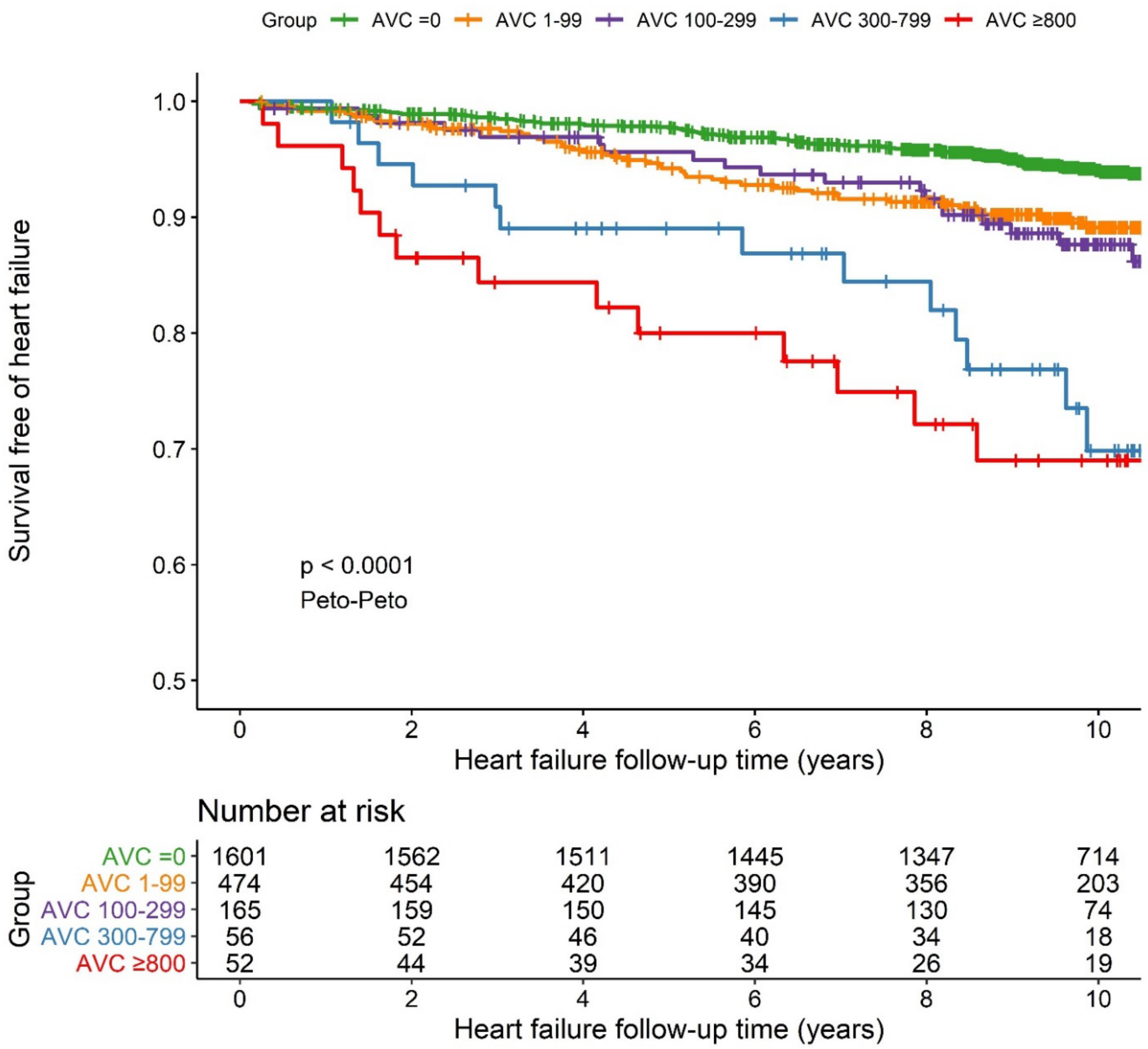

Figure S1. Kaplan-Meier survival curves for incident heart failure according to aortic valve calcium categories. AVC = aortic valve calcium.

Supplemental Tables.

Table S1. Multivariable Cox proportional hazard regression analyses of the association between aortic valve calcium and incident heart failure

	Event/N	Model 1		Model 2	
		HR (95% CI)	*p* value	HR (95% CI)	*p* value
**Continuous scale**					
Per 1 unit increase in log (AVC+1)	-	1.13 (1.06, 1.20)	<0.001	1.10 (1.04, 1.17)	0.002
**Categorical scale**					
Presence *versus* absence of AVC					
No AVC present	88/1601	Reference	-	Reference	-
AVC present	94/747	1.53 (1.12, 2.08)	0.007	1.37 (1.01, 1.87)	0.048
AVC categories score					
=0	88/1601	Reference	-	Reference	-
1-99	47/474	1.30 (0.90, 1.87)	0.151	1.19 (0.82, 1.72)	0.365
100-299	20/165	1.36 (0.83, 2.25)	0.222	1.23 (0.75, 2.02)	0.417
300-799	13/56	3.17 (1.75, 5.73)	<0.001	2.35 (1.27, 4.35)	0.007
≥800	14/52	2.61 (1.42, 4.79)	0.002	2.54 (1.37, 4.70)	0.003

Model 1 was adjusted for age and sex; model 2 additionally included body mass index, smoking status, systolic blood pressure, blood pressure-lowering medication, diabetes, total and high-density lipoprotein cholesterol, statins therapy, history of atrial fibrillation, history of coronary heart disease and coronary artery calcium. AVC = aortic valve calcium, HR = hazard ratio.

Table S2. Multivariable linear regression analyses of the association between categories of aortic valve calcium with echocardiographic parameters of cardiac structure and function

Echocardiographic parameters	Number	Model 1		Model 2	
		β (95% CI)	*p* value	β (95% CI)	*p* value
Left ventricular mass indexed by BSA					
=0	1543	Reference	-	Reference	-
1-99	452	2.25 (0.35, 4.16)	0.021	1.46 (-0.40, 3.33)	0.125
100-299	151	2.99 (-0.02, 5.99)	0.051	1.57 (-1.38, 4.51)	0.297
300-799	50	4.76 (-0.25, 9.78)	0.063	3.03 (-1.87, 7.92)	0.225
≥800	49	24.37 (19.27, 29.47)	<0.001	22.01 (17.03, 26.99)	<0.001
Left atrium diameter					
=0	1557	Reference	-	Reference	-
1-99	456	0.07 (0.01, 0.12)	0.018	0.06 (0.01, 0.11)	0.038
100-299	158	0.06 (-0.02, 0.15)	0.147	0.02 (-0.06, 0.1)	0.656
300-799	51	0.10 (-0.04, 0.25)	0.166	0.05 (-0.09, 0.18)	0.520
≥800	51	0.25 (0.10, 0.40)	0.001	0.17 (0.03, 0.31)	0.019
Cardiac output indexed by BSA					
=0	1538	Reference	-	Reference	-
1-99	447	-0.01 (-0.09, 0.06)	0.696	-0.01 (-0.09, 0.07)	0.795
100-299	151	-0.06 (-0.18, 0.06)	0.330	-0.03 (-0.15, 0.09)	0.606
300-799	50	0.07 (-0.13, 0.27)	0.480	0.09 (-0.11, 0.29)	0.369
≥800	50	0.05 (-0.15, 0.25)	0.610	0.04 (-0.15, 0.24)	0.665
Left ventricular ejection fraction (%)					
=0	1544	Reference	-	Reference	-
1-99	449	-1.0 (-1.80, -0.21)	0.014	-0.94 (-1.71, -0.17)	0.017
100-299	151	-0.87 (-2.13, 0.38)	0.174	-0.17 (-1.38, 1.04)	0.785
300-799	51	-0.05 (-2.12, 2.02)	0.962	0.81 (-1.19, 2.81)	0.426
≥800	50	-1.65 (-3.76, 0.46)	0.126	-1.31 (-3.34, 0.73)	0.208
E/A ratio					
=0	1533	Reference	-	Reference	-
1-99	449	-0.01 (-0.04, 0.02)	0.412	-0.01 (-0.04, 0.02)	0.565
100-299	152	-0.04 (-0.09, 0.01)	0.083	-0.04 (-0.08, 0.01)	0.136
300-799	50	-0.06 (-0.14, 0.01)	0.110	-0.06 (-0.14, 0.02)	0.140
≥800	48	-0.02 (-0.10, 0.06)	0.691	-0.01 (-0.09, 0.07)	0.859

Model 1 was adjusted for age and sex; model 2 additionally included body mass index, smoking status, systolic blood pressure, blood pressure-lowering medication, diabetes, total and high-density lipoprotein cholesterol, statins therapy, history of atrial fibrillation, history of coronary heart disease and coronary artery calcium. BSA = body surface area.

Table S3. Multivariable competing risk regression analyses of the association between aortic valve calcium and mortality

	Event/N	Model 1		Model 2	
		sHR (95% CI)	*p* value	sHR (95% CI)	*p* value
**Continuous scale**					
Per 1 unit increase in log (AVC+1)	-	1.01 (0.97, 1.06)	0.540	1.01 (0.97, 1.06)	0.510
**Categorical scale**					
Presence *versus* absence of AVC					
No AVC present	291/1601	Reference	-	Reference	-
AVC present	265/747	1.19 (0.98, 1.44)	0.083	1.11 (0.92, 1.35)	0.280
AVC categories score					
=0	291/1601	Reference	-	Reference	-
1-99	157/474	1.26 (1.02, 1.56)	0.033	1.18 (0.95, 1.47)	0.140
100-299	53/165	1.00 (0.72, 1.40)	1.000	0.95 (0.68, 1.34)	0.770
300-799	29/56	1.32 (0.78, 2.23)	0.300	1.11 (0.66, 1.87)	0.700
≥800	26/52	1.01 (0.57, 1.77)	0.980	1.03 (0.59, 1.80)	0.920

Model 1 was adjusted for age and sex; model 2 additionally included body mass index, smoking status, systolic blood pressure, blood pressure-lowering medication, diabetes, total and high-density lipoprotein cholesterol, statins therapy, history of atrial fibrillation, history of coronary heart disease and coronary artery calcium. AVC = aortic valve calcium, sHR = subdistribution hazard ratio.

Table S4. Multivariable competing risk regression analyses of the association between aortic valve calcium and incident heart failure among participants free of cardiovascular disease at baseline

	Event/N	Model 1		Model 2	
		sHR (95% CI)	*p* value	sHR (95% CI)	*p* value
**Continuous scale**					
Per 1 unit increase in log (AVC+1)	-	1.14 (1.05, 1.24)	0.002	1.11 (1.03, 1.21)	0.009
**Categorical scale**					
Presence *versus* absence of AVC					
No AVC present	62/1473	Reference	-	Reference	-
AVC present	63/627	1.68 (1.15, 2.45)	0.007	1.52 (1.04, 2.21)	0.029
Multi-categorical AVC score					
=0	62/1473	Reference	-	Reference	-
1-99	36/415	1.55 (1.01, 2.37)	0.045	1.42 (0.93, 2.17)	0.100
100-299	11/127	1.34 (0.70, 2.58)	0.380	1.22 (0.63, 2.37)	0.560
300-799	6/43	2.40 (1.00, 5.72)	0.049	2.11 (0.88, 5.09)	0.096
≥800	10/42	3.54 (1.67, 7.50)	<0.001	2.84 (1.34, 6.05)	0.006

Mortality was treated as a competing risk. Model 1 was adjusted for age and sex; model 2 additionally included body mass index, smoking status, systolic blood pressure, blood pressure-lowering medication, diabetes, total and high-density lipoprotein cholesterol, statins therapy, and coronary artery calcium. AVC = aortic valve calcium, sHR = subdistribution hazard ratio.

Table S5. Multivariable competing risk regression analyses of the association between aortic valve calcium and incident heart failure among participants without aortic valve stenosis

	Event/N	Model 1		Model 2	
		sHR (95% CI)	*p* value	sHR (95% CI)	*p* value
**Continuous scale**					
Per 1 unit increase in log (AVC+1)	-	1.09 (1.01, 1.17)	0.022	1.06 (0.99, 1.14)	0.100
**Categorical scale**					
Presence *versus* absence of AVC					
No AVC present	88/1600	Reference	-	Reference	-
AVC present	83/711	1.38 (0.98, 1.94)	0.059	1.22 (0.87, 1.71)	0.240
Multi-categorical AVC score					
=0	88/1600	Reference	-	Reference	-
1-99	47/473	1.24 (0.85, 1.82)	0.270	1.11 (0.75, 1.66)	0.600
100-299	19/160	1.36 (0.81, 2.31)	0.250	1.27 (0.76, 2.13)	0.360
300-799	11/48	2.77 (1.45, 5.29)	0.002	2.25 (1.19, 4.22)	0.012
≥800	6/30	1.56 (0.65, 3.78)	0.320	1.47 (0.59, 3.65)	0.410

Mortality was treated as a competing risk. Model 1 was adjusted for age and sex; model 2 additionally included body mass index, smoking status, systolic blood pressure, blood pressure-lowering medication, diabetes, total and high-density lipoprotein cholesterol, statins therapy, history of atrial fibrillation, history of coronary heart disease and coronary artery calcium. AVC = aortic valve calcium, sHR = subdistribution hazard ratio.

Table S6. Multivariable competing risk regression analyses of the association between aortic valve calcium and incident heart failure excluding participants who developed coronary heart disease prior to onset heart failure

	Event/N	Model 1		Model 2	
		sHR (95% CI)	*p* value	sHR (95% CI)	*p* value
**Continuous scale**					
Per 1 unit increase in log (AVC+1)	-	1.09 (1.02, 1.17)	0.011	1.08 (1.01, 1.16)	0.031
**Categorical scale**					
Presence *versus* absence of AVC					
No AVC present	77/1590	Reference	-	Reference	-
AVC present	74/727	1.31 (0.94, 1.84)	0.113	1.22 (0.87, 1.71)	0.261
AVC categories score					
=0	77/1590	Reference	-	Reference	-
1-99	39/466	1.17 (0.78, 1.77)	0.450	1.06 (0.69, 1.62)	0.800
100-299	16/161	1.26 (0.72, 2.23)	0.420	1.19 (0.68, 2.09)	0.540
300-799	6/49	1.67 (0.72, 3.88)	0.240	1.82 (0.67, 3.80)	0.290
≥800	13/51	2.54 (1.26, 5.11)	0.009	2.30 (1.19, 4.88)	0.015

Mortality was treated as a competing risk. Model 1 was adjusted for age and sex; model 2 additionally included body mass index, smoking status, systolic blood pressure, blood pressure-lowering medication, diabetes, total and high-density lipoprotein cholesterol, statins therapy, history of atrial fibrillation, history of coronary heart disease and coronary artery calcium. AVC = aortic valve calcium, sHR = subdistribution hazard ratio.
